# Multiple Lumbar Compression Fractures Following a New-Onset Seizure: A Case Report

**DOI:** 10.7759/cureus.99545

**Published:** 2025-12-18

**Authors:** Ruilin Wang, Serena Votapek, Jessica F Okun

**Affiliations:** 1 College of Osteopathic Medicine, Nova Southeastern University Dr. Kiran C. Patel College of Osteopathic Medicine, Davie, USA; 2 Department of Neurosurgery, Nova Southeastern University Dr. Kiran C. Patel College of Osteopathic Medicine, Davie, USA

**Keywords:** ballon kyphoplasty, lumbar spine, seizure-induced spinal fractures, tonic-clonic seizure, vertebral compression fractures

## Abstract

Vertebral compression fractures (VCFs) occur when axial forces exceed the vertebra’s strength, such as those occurring with low-impact injuries in osteoporotic patients. In healthy individuals, VCFs require significant trauma. Though rare, seizures can cause spinal compression due to muscle contractions. These fractures often affect the mid-thoracic spine and are usually asymptomatic due to the postictal state. This case discusses an uncommon incidence of multiple lumbar fractures following a seizure, otherwise known as seizure-induced spinal fractures. A 50-year-old male with a past medical history of hypertension, type 2 diabetes, and obesity presented to the emergency room (ER) after being found unresponsive following an unwitnessed seizure. The patient experienced a witnessed generalized tonic-clonic seizure in the ER with associated bladder incontinence. CT of the head was performed to rule out an acute cerebrovascular accident, and he was later discharged home with levetiracetam. The patient returned to the ER three days later with severe lower back pain, and a CT of the lumbar spine found compression fractures at L1, L4, and L5. Neurosurgery was consulted and subsequently performed kyphoplasty. Follow-up evaluation showed recovery with no reported pain or neurological deficits. Clinicians should maintain a high index of suspicion for compression fractures in the lumbar spine after a patient experiences a new-onset convulsive seizure, especially for those with risk factors.

## Introduction

Vertebral compression fractures (VCFs) are the result of an axial force that exceeds the structural integrity of the vertebra. A low-impact injury may be sufficient to cause VCFs in osteoporotic patients, and VCFs are the most common osteoporotic fracture. However, in a patient with normal bone density, VCFs are traumatic fractures that require substantial force to occur [1]. While uncommon, spinal compression fractures following seizures are attributed to violent muscle contractions, and seizure-induced spinal fractures (SISF) have an incidence rate of 0.04% [2,3]. The mechanism of this injury involves simultaneous forward flexion of the spine and paraspinal muscle contraction, primarily occurring during the tonic phase [4,5]. Many spinal injuries following a seizure are asymptomatic, often due to the patient’s altered mental status during the postictal period. Compression fractures have a higher prevalence than burst fractures, typically involving the mid-thoracic spine from segments T3-T8 [5]. A first seizure in a patient with no prior brain trauma or family history of epilepsy is often attributed to metabolic disturbances involving blood glucose, sodium, calcium, and magnesium. However, an isolated seizure may occur in the absence of an identifiable trigger [6]. The following case demonstrates the rare finding of a seizure-induced fracture of the lumbar spine in a non-osteoporotic patient with no prior history of seizure disorder. This novel finding highlights the importance of evaluating for spinal injury following a single seizure episode in patients with persistent pain or with notable risk factors.

## Case presentation

A 50-year-old morbidly obese male (body mass index: 40.1 kg/m²) with a past medical history of chronic essential hypertension, atrial flutter, and type 2 diabetes mellitus presented to the emergency department in a postictal state following an unwitnessed seizure. He was found unresponsive by his roommate and was transported to the emergency department by emergency medical services. The patient experienced another seizure upon arrival and was admitted for further management. CT of the brain showed no acute cerebrovascular events, and electroencephalography (EEG) conducted on the day of admission did not demonstrate any epileptiform activity. Admission labs were significant for an elevated non-fasting blood glucose of 337 mg/dL. Neurological examination was non-focal following the patient’s postictal state, but the patient was unable to provide pertinent information regarding the event due to his clinical condition. He denied any history of seizure disorder or osteoporosis, and initial treatment included 2 mg of intravenous lorazepam and 1,000 mg of intravenous levetiracetam.

The patient was discharged with levetiracetam 500 mg, aspirin 81 mg, and no imaging of the lumbar spine. Aspirin was prescribed due to his comorbidities of diabetes mellitus and hypertension, which resulted in a CHA₂DS₂-VASc score of 2, an indication for antithrombotic therapy [7].

**Figure 1:**
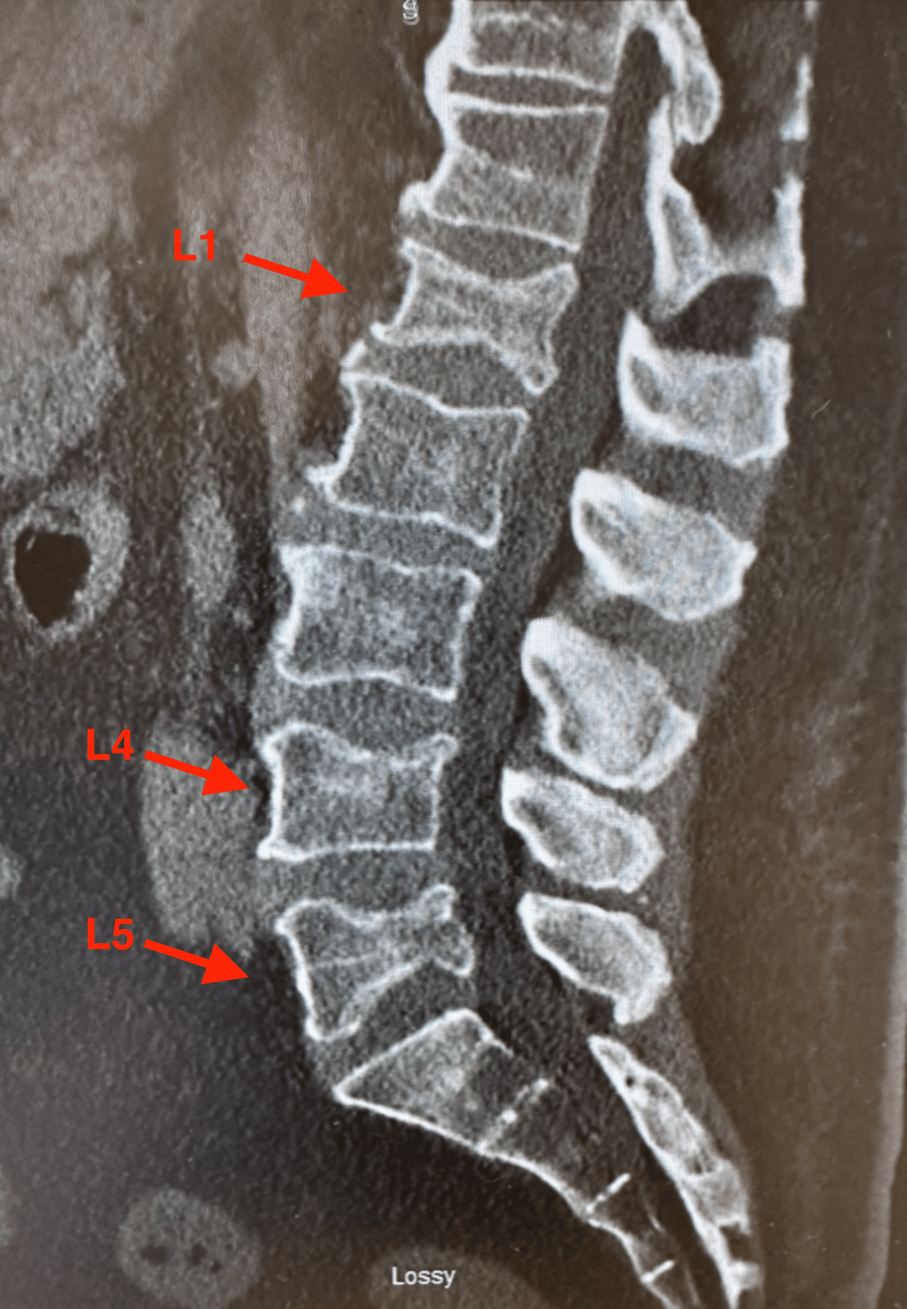
Sagittal CT scan of the lumbar spine demonstrating compression deformities involving L1, L4, and L5 (red arrows). The vertebral bodies show anterior wedging, decreased height, and cortical irregularity consistent with acute to subacute compression fractures. Image captured using a smartphone camera due to limitations in accessing downloadable files. All identifiable information was removed.

**Figure 2:**
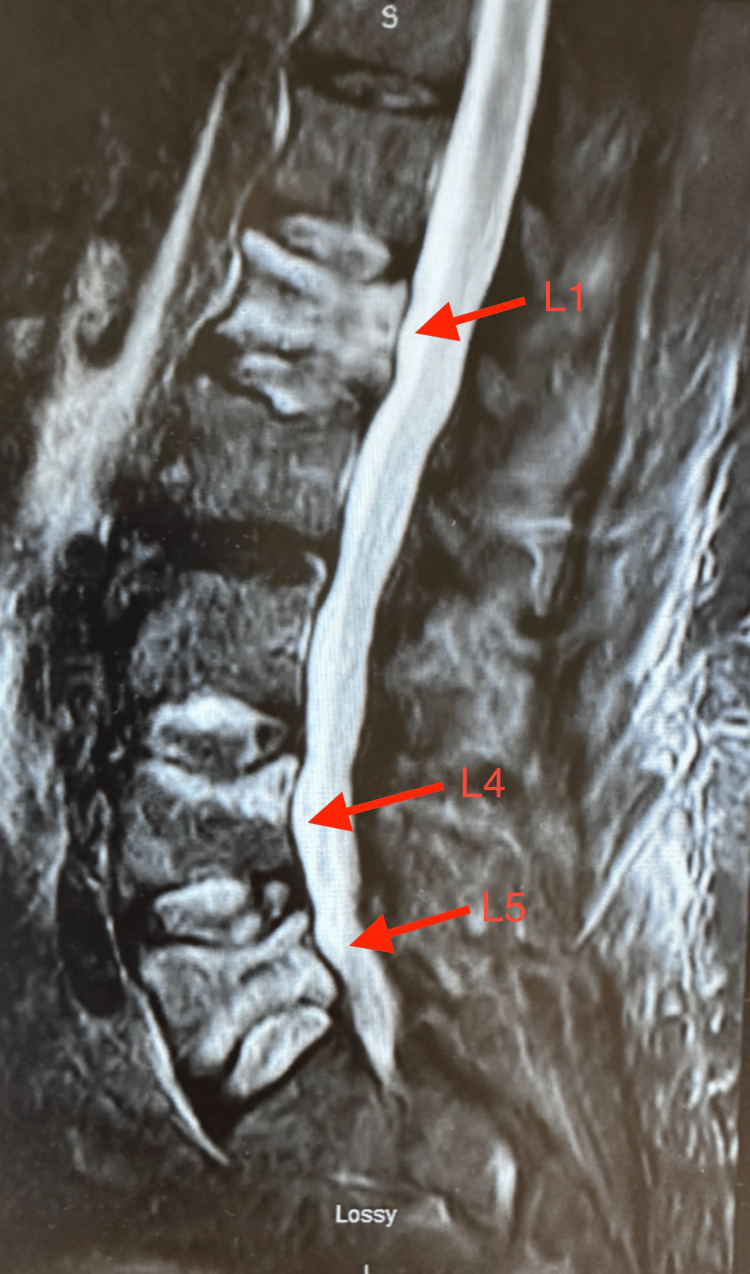
Sagittal T2-weighted MRI of the lumbar spine demonstrating hyperintense marrow edema at L1, L4, and L5 (red arrows). Vertebral bodies are consistent with acute to subacute compression fractures. Image captured using a smartphone camera due to limitations in accessing downloadable imaging files. All identifiable information was removed.

**Figure 3:**
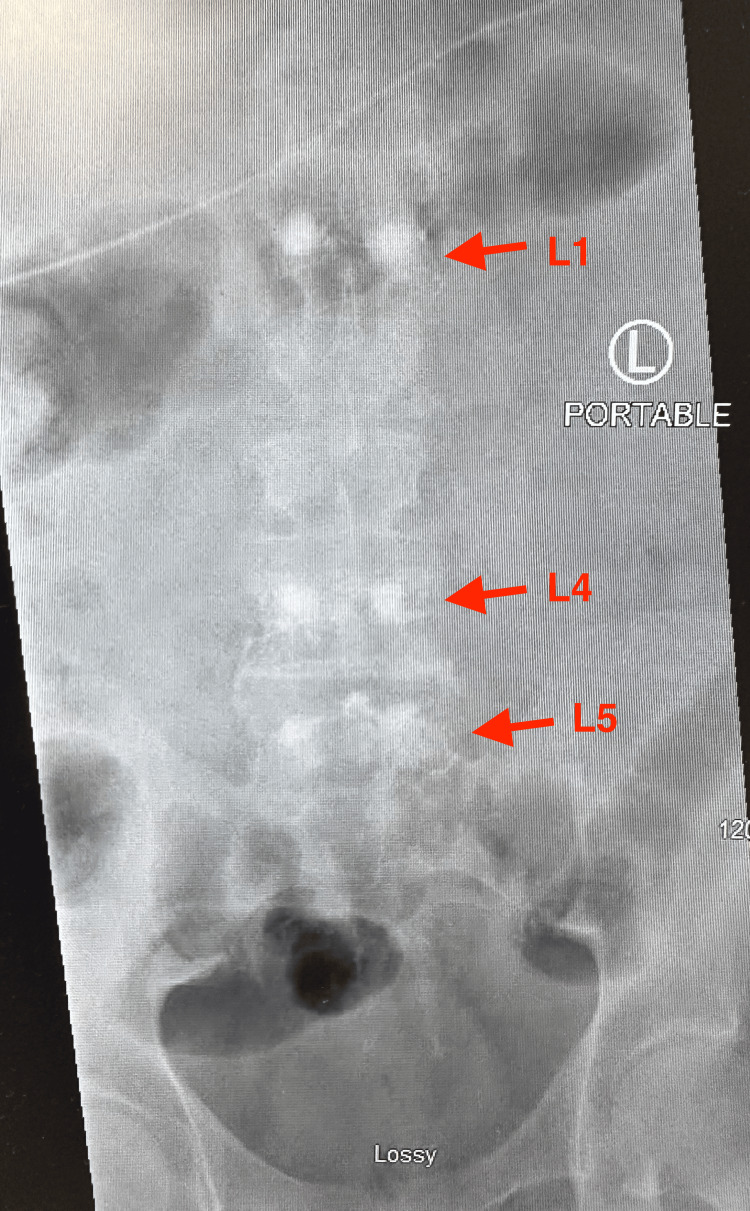
Portable anteroposterior lumbar spine X-ray demonstrating increased radiodensity consistent with compression deformities at L1, L4, and L5 (red arrows). Images captured using a smartphone camera due to limitations in accessing downloadable files. All identifiable information was removed.

**Figure 4:**
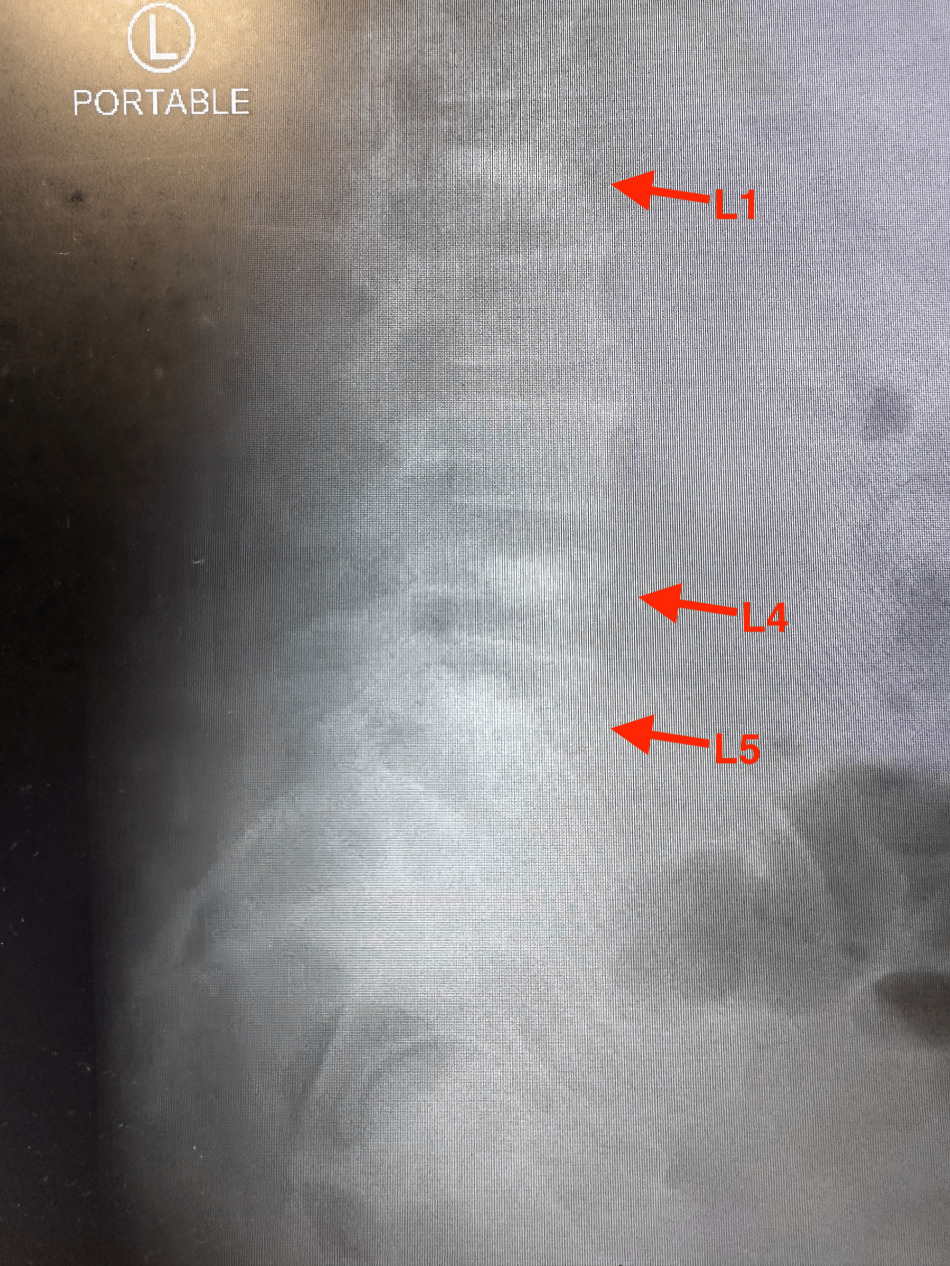
Portable lateral lumbar spine X-ray demonstrating compression deformities at L1, L4, and L5 (red arrows). Images captured using a smartphone camera due to limitations in accessing downloadable files. All identifiable information was removed.

Three days later, the patient was admitted back to the emergency department due to severe bilateral lower back pain. Sagittal CT imaging of the lumbar spine obtained during the second admission revealed acute to subacute compression injuries of L1, L5, and the superior endplate of L4 (Figure 1). Subsequent sagittal T2-weighted MRI of the lumbar spine demonstrated acute paraspinal soft-tissue edema and compression deformities at L1, L4, and L5, without evidence of spinal canal stenosis (Figure 2). Portable radiographs further showed increased radiodensity at these same levels, consistent with compression deformities (Figures 3, 4).

**Figure 5:**
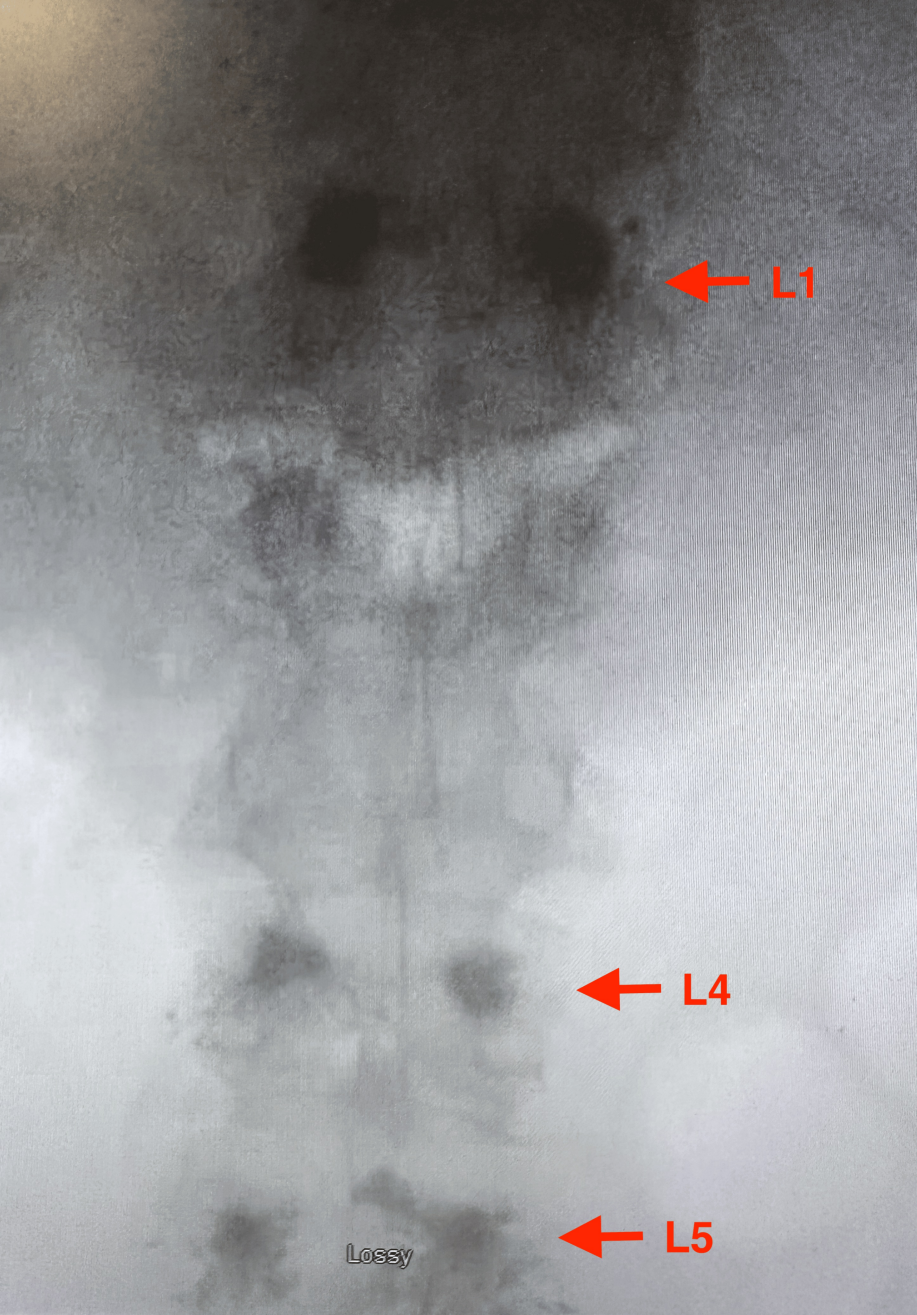
Postoperative anteroposterior lumbar spine radiograph demonstrating radiopaque kyphoplasty cement within treated vertebral bodies L1, L4, and L5 (red arrow). The image appears enlarged due to portable bedside acquisition. Image captured using a smartphone camera due to limitations in accessing downloadable imaging files. All identifiable information was removed.

**Figure 6:**
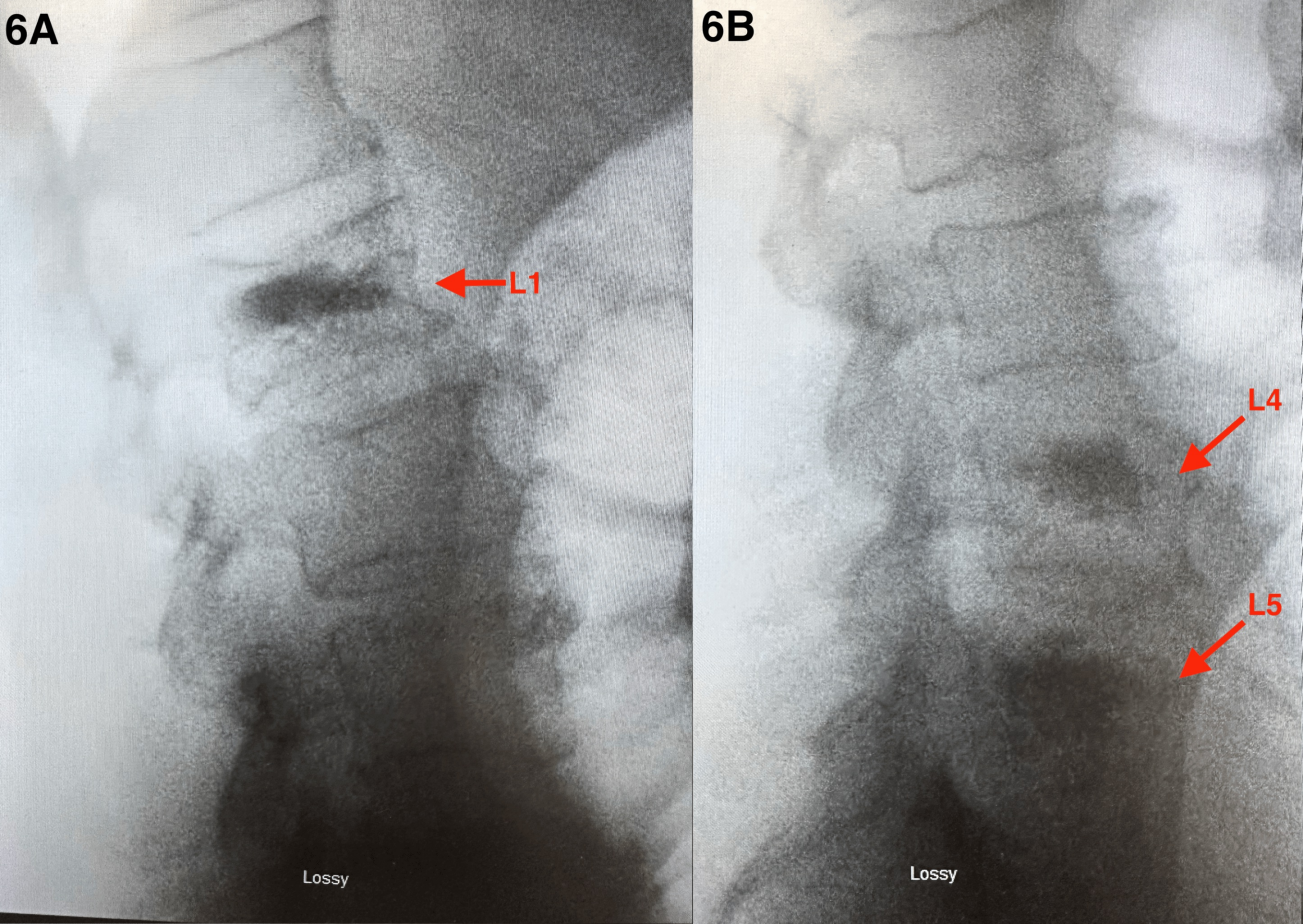
Postoperative lateral lumbar spine radiographs demonstrate vertebral body augmentation at L1 (A), L4, and L5 (B) with increased radiodensity consistent with kyphoplasty cement placement. Restoration of vertebral body height is noted without evidence of cement extravasation, posterior wall retropulsion, or spinal canal compromise. Findings are consistent with post-procedural stabilization of vertebral compression fractures. Image captured using a smartphone camera due to limitations in accessing downloadable imaging files. All identifiable information was removed.

Upon neurosurgery consultation, cement augmentation via balloon kyphoplasty was performed on L1, L4, and L5. Balloon kyphoplasty was pursued due to its significant increase in pain reduction and disability improvement compared to a conservative approach [8]. A postoperative portable anteroposterior radiograph showed radiopaque kyphoplasty cement appropriately placed within the treated vertebral bodies, confirming successful augmentation (Figure 5), and was confirmed with lateral lumbar spine radiographs (Figures 6A, 6B). Post-kyphoplasty, the patient’s pain improved to near resolution with no new reported seizures. At the two-week outpatient follow-up, physical examination showed intact cranial nerves, full (5/5) strength in lower extremities, symmetric 2+ reflexes, steady gait, and no focal neurologic deficits.

## Discussion

SISFs are an uncommon complication of generalized tonic-clonic seizures. The proposed mechanism involves violent paraspinal muscle contractions generating sufficient forces to cause vertebral compression fractures in the absence of external trauma [4]. Most compression fractures from seizures are unreported, especially when patients do not experience immediate symptoms such as back pain or paraparesis [2]. When reported, the site most commonly involved in SISFs is the upper lumbar spine (L1-L3) and mid-thoracic spine (T5-­T8) due to the natural kyphosis and mechanical vulnerability of this region [2].

This case differs from previously reported SISF patterns in both anatomic distribution and fracture morphology. While L1 fractures are commonly reported, involvement of the lower lumbar spine (L4, L5) is rare in the literature [2]. In addition, the L4 injury in this patient demonstrated features consistent with a possible AO Spine A3 burst fracture, a pattern more commonly associated with high-energy trauma rather than seizure activity alone [2]. The patient’s presentation with absence of osteoporosis, a prior seizure disorder, or any clear trauma further supports the diagnosis of a seizure-induced mechanism.

Our case demonstrates how SISFs can be missed or diagnosed late. After a seizure, the initial workup often focuses on neurologic and metabolic cases, and spinal imaging may be overlooked without focal neurologic deficits or clear trauma. As lumbar spine imaging was not performed during the first hospital encounter, the diagnosis of vertebral fractures was delayed. Persistent or worsening back pain after a seizure should prompt consideration of spinal imaging, even in patients without known bone disease. Balloon kyphoplasty was performed due to persistent severe pain and imaging evidence of instability, in the absence of neurologic compromise or canal stenosis [9]. The patient’s rapid improvement after surgery further supports the conclusion that the compression fractures were the primary source of his pain and that operative management was effective.

## Conclusions

Our case highlights the rare occurrence of SISFs involving the lower lumbar spine (L4-L5), including features concerning an AO Spine A3 burst fracture, an injury pattern rarely reported in association with seizure activity alone. It also illustrates how spinal fractures may be missed or diagnosed late in the postictal setting, as early evaluation often focuses on neurologic and metabolic causes. Ongoing back pain following a seizure should prompt consideration of spinal imaging, even in patients without known bone disease. In this patient, delayed diagnosis led to continued pain, which improved rapidly after balloon kyphoplasty, confirming the fractures as the source of symptoms. This case serves as a reminder for clinicians to remain vigilant for the possibility of seizure-induced spinal fractures. It highlights the importance of a thorough physical assessment and imaging in postictal patients when there is associated back pain. This is especially important in those with risk factors, such as obesity, to avoid missed diagnoses and delayed necessary surgical intervention.
